# Predictive diagnosis of the cancer prone Li–Fraumeni syndrome by accident: new challenges through whole genome array testing

**DOI:** 10.1136/jmg.2008.064972

**Published:** 2009-03-05

**Authors:** T Schwarzbraun, A C Obenauf, A Langmann, U Gruber-Sedlmayr, K Wagner, M R Speicher, P M Kroisel

**Affiliations:** 1 Institute of Human Genetics, Medical University of Graz, Graz, Austria; 2 Department of Ophthalmology, Medical University of Graz, Graz, Austria; 3 Department of Neuropediatrics, Medical University of Graz, Austria

## Abstract

**Background:**

Li–Fraumeni syndrome greatly increases the risk of developing several types of cancer and is usually caused by *TP53* germline mutations. Predictive testing of at-risk family members is only offered after a complex genetic counselling process. Recently the clinical implementation of array comparative genomic hybridisation (CGH) has revolutionised the diagnosis of patients with syndromic or non-syndromic mental retardation and has evolved to a routinely performed high resolution whole genome scan.

**Methods and results:**

When using array CGH to identify the cause for mental retardation in a 7-year-old child we found a submicroscopic de novo deletion of chromosome 17p13.1, which includes several genes likely to be causative for her phenotype, and also of *TP53*.

**Conclusion:**

Thus, array CGH resulted in an unintended predictive diagnosis of an increased tumour susceptibility as observed in Li–Fraumeni syndrome.

The list of well defined inherited cancer predisposition syndromes, which can be attributed to a hereditary susceptibility and have far reaching implications for all family members, is steadily growing. Many of these syndromes are caused by germline alterations in a tumour suppressor gene. Usually one functional copy of a tumour suppressor gene is sufficient to exert the function. However, inactivation of both alleles by mutation or deletion can result in uncontrolled proliferation and may therefore contribute to tumorigenesis. Hence, individuals who have already one dysfunctional tumour suppressor gene copy in all somatic cells due to a germline mutation may have such an increased tumour risk whenever the function of the second copy is compromised in a cell. This mechanism is usually referred to as Knudson’s “two-hit hypothesis”.^1^

In familial cancer syndromes the identification of at-risk family members by predictive testing is often recommended as enhanced surveillance for early diagnosis and prevention of disease is a critical part of primary care. Due to the extensive consequences of such predictive testing it is usually only offered after a complex genetic counselling process. Sophisticated surveillance guidelines with proven benefit were developed for several cancer prone syndromes such as Lynch syndrome (hereditary non-polyposis colon cancer (HNPCC)),^2^ hereditary breast and ovarian cancer due to mutations in *BRCA1* or *BRCA2*,^3^ or for hereditary childhood tumours such as retinoblastoma.^4^ ^5^

Array comparative genomic hybridisation (CGH) has evolved to a standard application in clinical genetics, especially in individuals with syndromic or non-syndromic mental retardation. As a consequence high resolution scans of entire genomes for small gains and losses anywhere in the genome are now conducted routinely. However, the commonly applied execution of array CGH bears the risk that deletions of genes involved in hereditary cancer syndromes, especially tumour suppressor genes, may also be identified, which would then result in an unintended predictive diagnosis of a cancer prone syndrome. Here we report such a case in which we identified a submicroscopic de novo deletion of chromosome 17p13.1, which included among other genes the tumour suppressor *TP53* gene. This scenario represents new challenges for both clinical oncologists and genetic counsellors.

## CASE REPORT

The proband was born at 42 weeks by spontaneous vaginal delivery following an uncomplicated pregnancy; the Apgar score was 7/9/10. Birth weight was 4180 g (>90th centile), length was 52 cm (75th centile), and the head circumference (OFC) was 37 cm (90th centile).

Examinations at 4 and 5 months revealed a psychomotor retardation with a generalised muscle hypotonia. Dysmorphic features included a broad and low set nasal bridge, a short philtrum and a bifid uvula. The child could not establish visual contact. Brain magnetic resonance imaging (MRI) showed the presence of an enlarged fourth ventricle, hypoplasia of the cerebellar vermis and corpus callosum, anomalies usually classified as incomplete manifestation of a Dandy–Walker malformation or Dandy–Walker variant. Within the first year short episodes of myoclonic seizures occurred, which could be prevented by administration of valproic acid and lamotrigine.

At 18 months of age she was able to sit. A severe bilateral visual impairment and a high myopia of −12 D were diagnosed.

At 7 years of age she was able to walk without support but with ataxic movements. She spoke a few words. At this age the patient was presented to our counselling service. In order to clarify the cause of her impairment we obtained informed consent from the parents to perform cytogenetic analysis and high resolution array CGH.

## METHODS

### Cytogenetic analysis

Chromosome banding analyses of the proposita and her parents were done according to standard protocols.

### Array CGH

Array CGH was carried out using a whole genome oligonucleotide microarray platform (Human Genome CGH 44B Microarray Kit; Agilent Technologies, Santa Clara, California, USA). This array consists of approximately 43 000 60-mer oligonucleotide probes with a spatial resolution of 43 kb. Samples were labelled with the Bioprime Array CGH Genomic Labeling System (Invitrogen, Carlsberg, California, USA) according to the manufacturer’s instructions. Further steps were performed according to the manufacturer’s protocol (version 6.0). Slides were scanned using a microarray scanner (G2505B) and images were analysed using CGH Analytics software 3.4.40 (both from Agilent Technologies) with the statistical algorithm ADM-2, sensitivity threshold was 6.0. At least three consecutive clones had to be aberrant to be identified as significant change.

### Fluorescence in situ hybridisation (FISH)

FISH was performed with a commercially available probe for the *TP53*-region (LSI p53 Abbott/Vysis) according to the manufacturer’s instructions.

### Quantitative real-time polymerase chain reaction (qPCR)

As previously described, qPCR was performed in order to verify the results from array CGH and to narrow down the breakpoint region.^6^ After narrowing down the breakpoint region to about 10 kb, we conducted PCR with primers from both sides. The junction fragment was subsequently sequenced and compared with the genomic sequence.

### Sequencing

RNA was isolated from peripheral blood using the PAXgene blood RNA system according to the manufacturer’s protocols (PreAnalytiX). For cDNA synthesis we used the Omniscript RT Kit (Qiagen) with oligo dT primers. Monoallelic expression of *GUCY2D* was determined by sequencing a single nucleotide polymorphism (SNP) (rs2816) from the 3′UTR of *GUCY2D* in both genomic DNA and cDNA.

Sequencing was performed by cycle sequencing using the ABI BigDye Terminator Cycle Sequencing Kit according to the supplier’s protocol and was analysed on an ABI3100 genetic analyser (both ABI).

## RESULTS AND DISCUSSION

**Figure 1:**
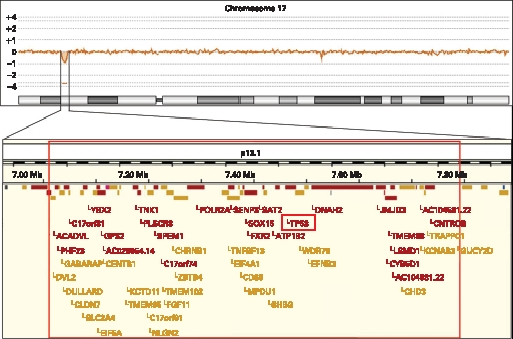
The top panel illustrates the array comparative genomic hybridisation profile of chromosome 17 demonstrating a small deletion in chromosome band 17p13.1. The lower panel depicts an enlargement of the deleted region. The exact localisation of the breakpoints was determined by sequence analysis. The *TP53* gene is almost at the centre of the deleted region. The *GUCY2D* gene is not included in the deleted region. However, the cis-regulatory elements are deleted, which explains the monoallelic expression of the gene and as a consequence the patient’s cone–rod dystrophy 6.

**Figure 2:**
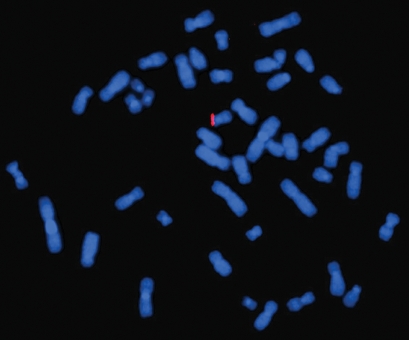
Hybridisation of a commercially available *TP53* specific probe to metaphase spreads of the proposita, resulting in only one signal confirming the deletion of the respective region.

**Figure 3:**
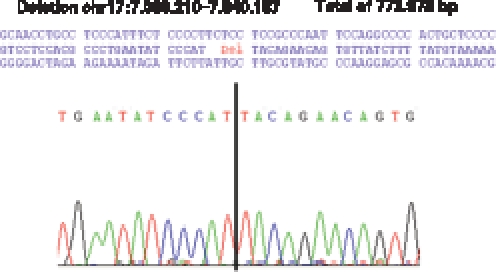
Chromatogram showing the exact location of the breakpoints and the resulting size of the deletion.

Standard banding analysis of the proposita showed a normal female karyotype (46,XX). In a next step we performed array CGH, which revealed a small deletion in chromosome 17p13.1 (fig 1). When analysing the DNA of the parents and their daughter by real-time PCR we identified this deletion only when using the daughter’s but not the parental DNA. Provided that none of the parents has a mosaic constellation the deletion most likely occurred de novo. Furthermore, we applied a commercially available *TP53*-specific probe to metaphase spreads of the proposita, which confirmed the deletion (fig 2). In addition, we determined the deletion size and the localisation of the breakpoints by sequencing (fig 3). In summary, the exact size of the deletion is 774 kb and contains 47 genes according to current database entries (Ensemble release 50; www.ensembl.org).

Several of the deleted genes can presumably be related to some of the patient’s phenotypic features. For example, loss of the *KCNAB3* potassium channel gene is likely to be involved in the occurrence of the patient’s seizures. Furthermore, the deletion includes several neurotransmitter genes and kinases, which may represent a contributing factor to the mental retardation. A prime example for phenotype–genotype correlation is the *GUCY2D* gene, which was disrupted from the cis-regulatory elements by the breakpoint. We verified monoallelic expression by sequencing an SNP (rs2816) within *GUCY2D* in both genomic DNA and cDNA. This resulted in cone–rod dystrophy 6 (CORD6; OMIM 601777), a diagnosis which was indeed confirmed by our ophthalmologists and explains our patient’s severely impaired vision.

However, we also identified a loss of the *TP53* gene within the deleted region. *TP53* regulates the cell cycle and functions as a tumour suppressor involved in preventing cancer. As such, *TP53* has been designated as the “guardian of the genome”.^7^ In fact, the *TP53* gene is the most commonly mutated gene in human cancer.^8^ The overall lifetime risk of cancer in patients with *TP53* germline mutations is in the range of 80–90%, with a risk as high as 40% within the first two decades of life.^9^

This condition was named Li–Fraumeni syndrome after the physicians who first recognised it.^10^ The classical definition of the Li–Fraumeni syndrome is based on the following three parameters: (1) a proband with a sarcoma diagnosed before the age of 45; (2) a first degree relative with cancer before the age of 45; and (3) another first or second degree relative with either a sarcoma diagnosed at any age or any cancer diagnosed under the age of 45.^11^ Although sarcomas are quite frequently observed in Li–Fraumeni syndrome, a broad range of other tumours may occur including breast cancer, bone cancer, brain tumours, lung cancer, laryngeal cancer, leukaemia, adrenal cortical neoplasia, and others. Furthermore, more relaxed criteria were released for variations of a Li–Fraumeni-like syndrome.^12^ ^13^

In the absence of a positive family history and without any tumour our patient does not formally fulfil the criteria for Li–Fraumeni or Li–Fraumeni-like syndrome. However, the complete loss of one copy of the *TP53* gene in our patient should result in a similar if not identical tumour risk as affected members of Li–Fraumeni syndrome families according to the aforementioned two-hit hypothesis. Therefore, we assess that our patient has a greatly increased tumour risk with all the unusual characteristics of the Li–Fraumeni syndrome, including that several kinds of cancers may be involved, that cancer may strike at a young age, and that cancer may strike several times throughout the patient’s life.

As mentioned before there are cancer prone syndromes in which clear guidelines for surveillance have been established and their benefit has been proven. In contrast, *TP53* germline mutations represent a special scenario, as the variable expressivity and penetrance and the diversity of the tumour spectrum render clinical surveillance and genetic testing a difficult task. As a consequence there are no guidelines for patients with *TP53* mutations and the benefit of any kind of surveillance has not been proven yet.^4^ ^14^ Nevertheless, the American Society of Clinical Oncology (ASCO) considers Li–Fraumeni syndrome to be a syndrome for which predictive testing should be considered, especially within research studies.^15^

However, these considerations usually refer to an adult individual who is able to give informed consent. In the case of children, who are not able to give informed consent, predictive *TP53* testing is controversial. Recommendations include that a multidisciplinary team should decide whether such a test for a cancer predisposition syndrome, in which many tumours do not manifest until later in life, is to the child’s benefit. Therefore, the welfare of the child should always be the major consideration.^5^

In our case such careful considerations before testing had not been possible. The patient’s situation is further complicated by her severe mental retardation, as she may complain about possible symptoms from a tumour growth only at an advanced stage or even not at all, which poses an additional risk for a delayed diagnosis. We informed the patient’s parents in extensive genetic counselling sessions. Furthermore, we formed a multidisciplinary team in order to discuss possible surveillance options. At present this surveillance is confined to regular physical examinations and ultrasound examinations of the abdomen.

As array CGH has evolved into a standard diagnostic tool for a variety of conditions,^16^ it is very likely that with the advancing use of this technology similar deletions, or deletions of other cancer prone syndromes, will be identified. In each case it will place the physician or genetic counsellor in a special situation as a risk evaluation for tumour development will have to be offered, although this had not been requested initially by the patient or her/his family. The growing risk of identifying individuals with an increased lifetime risk for cancer by accident will fuel the need for sophisticated risk stratification to achieve a better prediction for the occurrence of possible tumour types and the timing of cancer.^17^
